# The Interplay between Branching and Pruning on Neuronal Target Search during Developmental Growth: Functional Role and Implications

**DOI:** 10.1371/journal.pone.0025135

**Published:** 2011-10-20

**Authors:** Remus Oşan, Emily Su, Troy Shinbrot

**Affiliations:** 1 Department of Mathematics and Statistics, Georgia State University, Atlanta, Georgia, United States of America; 2 Department of Mathematics and Statistics, Boston University, Boston, Massachusetts, United States of America; 3 Department of Biomedical Engineering, Rutgers University, Piscataway, New Jersey, United States of America; University of Nebraska Medical Center, United States of America

## Abstract

Regenerative strategies that facilitate the regrowth and reconnection of neurons are some of the most promising methods in spinal cord injury research. An essential part of these strategies is an increased understanding of the mechanisms by which growing neurites seek out and synapse with viable targets. In this paper, we use computational and theoretical tools to examine the targeting efficiency of growing neurites subject to limited resources, such as maximum total neural tree length. We find that in order to efficiently reach a particular target, growing neurites must achieve balance between pruning and branching: rapidly growing neurites that do not prune will exhaust their resources, and frequently pruning neurites will fail to explore space effectively. We also find that the optimal branching/pruning balance must shift as the target distance changes: different strategies are called for to reach nearby vs. distant targets. This suggests the existence of a currently unidentified higher-level regulatory factor to control arborization dynamics. We propose that these findings may be useful in future therapies seeking to improve targeting rates through manipulation of arborization behaviors.

## Introduction

Search and pursuit problems have been studied extensively, leading to optimal strategies, for example search for food in an unpatterned landscape [1], pursuit of a duck in a circular pond [2] or search along persistent random walks [3]. In this paper, we study the search by neurons of viable targets during regeneration. As we will show, this problem presents particular issues that distinguish it from other historical search exercises – for instance, a single axon can branch to produce multiple search avenues, and an axon probing a fruitless avenue can die back, recovering cellular resources.

During normal development, controlled growth and elaboration of neuronal extensions (axons, dendrites, and synaptic connections) are central to establishing a functional nervous system [4]–[5]. Accordingly, deficits and alterations in the programmed neural architecture caused by trauma lead to impaired function. After spinal cord injury, for example, injury to both the central nervous system (CNS) and peripheral nervous system (PNS) lead to loss of motor and sensory capabilities. Consequently, re-establishing functional connections is essential to successful post-traumatic repair of the nervous system.

Axonal connectivity between neurons is complex and varied, involving morphologies that facilitate connections to very different types of targets [6]–[7]. For many neurons, this means that exuberant axonal projections generated during development must be differentially regulated so that beneficial branches are elongated while aberrant branches are eliminated [8]–[9]. For other neurons, direct, unwavering axonal trajectories are abruptly and purposefully eliminated after their collaterals have reached an appropriate target. This large scale axon degeneration has been documented and studied in a variety of developmental systems, for example in retinotopic mapping in chick and the superior colliculus of mice [10]. The relationship between neuronal morphology and synaptic connectivity is exemplified by the heterogeneous population of neurons found in the dorsal root ganglion (DRG), where complexity and variability in geometric shapes of sensory neurons are observed, thus reflecting the diverse range of modalities served by DRG neurons [11]. In the present work, we focus on one piece of this puzzle: the interplay between branching and branch-elimination processes in establishing appropriate synaptic partnerships.

Mathematical and computational studies within the field of neuroscience have previously been used to examine the spatio-temporal organization of post-synaptic potentials within a dendritic network. For example, quantitative models of the detailed branching patterns in dendritic trees have investigated the impact of network topology on firing patterns and neuronal signal processing [12]–[15]. Several modeling approaches have been used in the *in silico* synthesis of dendritic trees as well. These can be characterized either as Growth Models or Reconstruction Models. Growth Models are based on principles of dendritic development, utilizing rules of outgrowth associated with dynamic growth-cone behavior, microtubule-mediated neurite elongation, and actin meshwork branch formation [16]–[19]. In contrast, Reconstruction Models use an algorithm based on a canonical set of elementary properties which are originally derived from characterizing an existing dendritic structure [20]–[21]. Although generated from minimalistic rules, the emergent arbor morphologies of the reproduced neurons are statistically indistinguishable from a sample of real neurons. Note that Reconstruction Modeling is a purely descriptive approach which uses minimal rules to “synthesize” topologically-realistic neurons. In contrast, Growth Modeling adopts an exploratory approach by using biological rules of development and observations of the outgrowth process to explain or predict variations in full-grown arbor structures [22]. This paper introduces a conceptually new approach to Growth Modeling by incorporating a pruning function into the algorithm and evaluating the growth of the neurons in the context of a target-search problem. In contrast to both growth and reconstructionist modeling which focus on the finalized structure of a neuron, our approach examines the evolution of a neuron through its time-steps of development and addresses the potential for its intermediate morphologies to establish connections. As a result, the focus or our research shifts from faithfully mimicking the neural structures obtained in within the *in vitro* experiments toward asking the question: how successful are neurons with similar growth properties in reaching their targets?

Two types of optimization strategies present themselves when context-dependent constraints are placed on a neuron during growth. In the first scenario, neurites may aim to reach targets in the shortest time possible. Strategies which minimize search time may be at work in certain developmental stages, e.g. during pyramidal [23] or optic [24] decussation where axons must cross the midline within a specified time window. In this paper, however, we focus on search strategies which are of significance to ongoing *in vitro* studies and potential future therapies involving adult CNS regeneration. For these purposes, time may be less of an issue than limitations in resources. In order to maximize the space explored under this constraint, a neuron that seeks to reach a target could branch as often as possible and prune as seldom as possible. In practice, however, this would create an arbor whose cumulative length of all of its branches would grow exponentially rapidly and which would at some point inevitably exhaust any cell's resources. In the present paper, therefore, we focus on optimal search strategies for a neuron with a fixed resource limitation; that is, for a neuron that produces an arbor with a specified maximum cumulative length.

The issue of neural connections across extended spatial scales has been also examined in depth in the context of neural wiring for the brain structures, such as hippocampus or cortex, [25]–[29]. In such situations, the overall neural structure is the result of a local optimization problem which seeks to minimize the associated metabolic costs at each branching point. We note here that the re-establishment of the communication pathways for spinal cord injury occurs at different spatial scales, and the resulting neural structures are significantly less compact as the ones from the brain regions mentioned in the context of the neural wiring research.

## Methods

### *In vitro* branching and pruning effects

**Figure 1:**
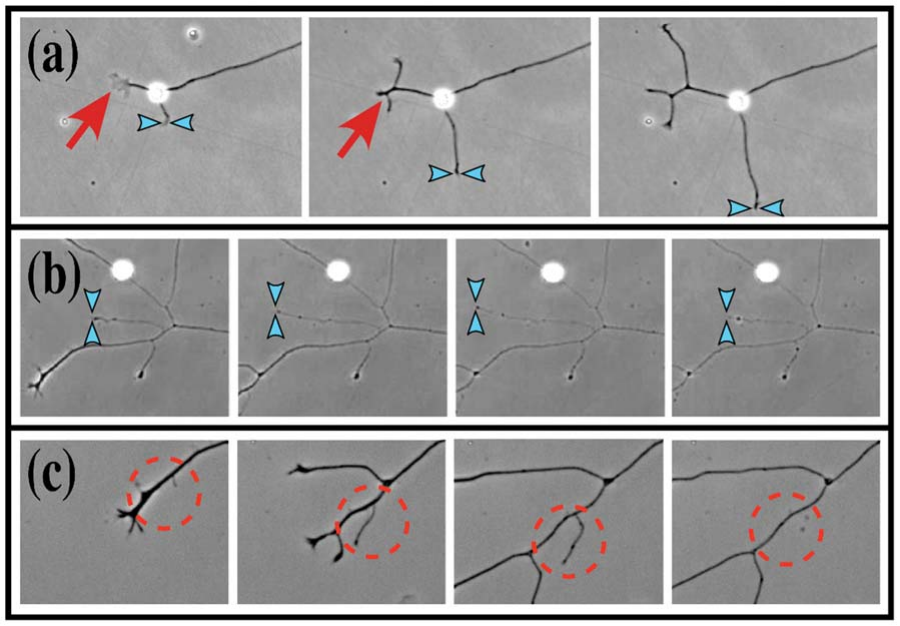
Time sequences showing branching and pruning of dissociated E11 chick dorsal root ganglion neurites. (a) Branching (red arrow) and extension (blue arrowheads) of primary axons. (b) Extension and retraction (blue arrowheads) of neurite tip. (c) Tertiary branching and pruning (encircled). Cultures are grown in the presence of glia in 5% CO_2_/ 37°C on Poly-L-lysine/laminin in N3 complete serum-free media. Phase-contrast live imaging at 28 hrs post-plating. Time interval between acquisitions for each time series is as follows: (a) 30 mins, (b) 75 mins, (c) 75 mins. Snapshots are contrast enhanced for visual clarity of the neurites.

Illustrative examples of both branching and branch elimination are shown in Fig. 1 from *in vitro* studies of Dorsal Root Ganglia were dissected from the lumbar region of embryonic chicks at day E11. Dissociated neurons and glial cells were isolated by digestion in 0.25% trypsin followed by mechanical trituration through a polished glass pipette and purification through a 10% BSA in PBS gradient. Neuro-glial suspensions were plated onto Poly-L-lysine/laminin coated plates and grown at 5% CO_2_/37°C in N3 complete serum-free media. Time-lapse movies of cocultures were acquired at 15 minute time intervals, 24–48 hours post-plating, with a 10× objective (N.A. 0.35) using an inverted Zeiss 200 M deconvolution microscope mounted with an on-stage incubation chamber and heating plate. Neurite tracing and morphometric analysis of live-cell phase contrast images were performed using ImageJ software. For each neuron, individual neurites were tagged, tracked, and traced across time frames. In the Figure 1, we show cases in which: a single neurite splits to form two or more secondary neurites (Fig. 1(a)), a growing neurite tip advances and then retracts (Fig. 1(b)), or branches are eliminated entirely over time (Fig. 1(c)). All of these processes will be discussed in detail in the *in silico* numerical simulations that follows, with a focus on evaluating how branching, advancement and retraction of neurite tips, as well as branch elimination affect axonal pathfinding and targeting strategies.

### Stochastic Model For Neurite Evolution

The simulation used here is straightforward, and involves elements that have been described elsewhere [30]. Intrinsically, neurites (a) grow stochastically out until a maximum total length of all branches in the arbor, L_max_, is achieved; (b) can bifurcate periodically with defined probability, P_branch_; and (c) are subject to pruning of available neurite tips with fixed probability, P_prune_. Each of these functions are described here.

#### (a) Neurite Growth

Growth begins at a fixed location, defining a cell ‘body’, and each neurite tip grows according to an integrated random walk [30], meaning that the neurite's velocity executes a random walk. Explicitly, the velocity of the i-th neurite tip at time t, V_i_(t) is assigned an interim value:
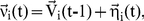
(1)where 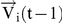 is the velocity one computational timestep earlier, and 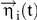 are vectors (one for each neurite tip) uniformly distributed in the entire 4π solid angle surrounding the origin, and with maximum amplitude 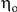. In principle, this interim velocity can grow without bound, whereas neurites have limited capacity for growth. We define the final velocity of the i-th tip to be bounded below a maximum value S_max_ as follows:
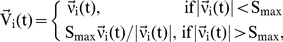
(2)In the simulations following, S_max_ = 1 for convenience, and 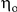 is taken to be 40% of S_max_.

#### (b) Branching

Branching of neurite tips occurs as follows. Every 10 computational timesteps, each neurite tip is permitted to split into two tips, with probability P_branch_ – this is accomplished by simply choosing a random number, r_i_, between 0 and 1 for each neurite tip, and if r_i_<P_branch_, a new tip is spawned. When a tip is spawned, this new tip is assigned a higher order than the prior tip, so the original neurite has order 1, its daughter has order 2, and so forth. When branching occurs, both parent and daughter have identical starting locations, but the newly-generated neurites acquire separate additional random additions to their velocities, of magnitudes amounting to 30%·S_max_. This addition gives the secondary branches a tendency to diverge, or in other words, to have a non-zero branching angle, as is seen in *in vitro* experiments (Figure 1). Each neurite tip, whether parent or daughter, continues to travel with velocity given by eq. [1], and is eligible to branch again after 10 further timesteps. We note here that in the real system, the assumption that the branching probability is fixed, is a simplification that permits us to employ analytical methods and to establish a baseline for the expected behavior. Future modifications to incorporate modulating factors in biological systems such as gradients in growth factors, complex boundaries, neuronal-glial interactions, etc. are desirable, but the first step of analyzing the dynamics underpinning these more complicated behaviors is the goal of the present study.

#### (c) Pruning

Every neurite tip is also subject to being pruned, with a fixed pruning probability, P_prune_. Pruning again occurs every 10 timesteps, and it is again determined algorithmically by assigning each tip a random number r_i_ between 0 and 1, and those tips with r_i_<P_prune_ are pruned. Explicitly, this means that the i^th^ tip is eliminated back to the nearest branch point, and is removed from the list of growing or branching neurites. Die-back beyond the nearest branch point does not occur in our model. Importantly, when pruning occurs, the length from the nearest branch point to the pruned tip is not counted against the total length of the tree – i.e. we assume that the resources associated with this tip are recovered by the neuron for further exploration.

### Target Search As An Optimization Problem

Before we examine the simulation results, we consider the theoretical constraints on neuronal targeting. We formulate the optimization problem as follows: given a neural tree of total constrained length L_max_, taken to be the sum of the lengths of all of its branches, what is the fixed branching probability P_branch_ that would maximize the number of search sites out to a radius, D, from the originating cell body? Here, the optimal neural tree is the one that maximizes the number of hits at the set distance D, which is equivalent to increasing the chance of success for finding a single target located at distance D away from the origin. Correspondingly, the optimal class of neurons is the set of neural trees generated with the same set of parameters that on average achieve maximal performances at distance D. To begin our investigation, we consider a simplified example to illustrate how the main parameters, especially branching and pruning probabilities, determine an optimal tree structure. To allow for analytical derivation of our results we will assume that: the neurites grow in a straight line, bifurcate at fixed time intervals and branch at angles are very close to zero, thus doubling the amount of search in the same spatial location, after each successful branching event.

## Results

### Expected Length of Tree Branches: An Analysis Derived from the Evolution of a Single Branch

We start by examining a neuron that does not prune any of its branches, for example a tree of maximum cumulative length L_max_ = 10 that searches for a target at distance D = 6 units of arbitrary length. We define the branching probability such that a given growing tip has the probability P_branch_ of bifurcating into two tips in a unit time, Δt, and consequently for growth tips that elongate at a constant rate, V_tip_, this in turn defines a mean distance traveled between two branching decision, L_0_, where L_0_ = V_tip_·Δt. Note that this distance is different from the average neurite length, L_average_ = V_tip_·Δt/P_branch_, as shown below.

**Figure 2:**
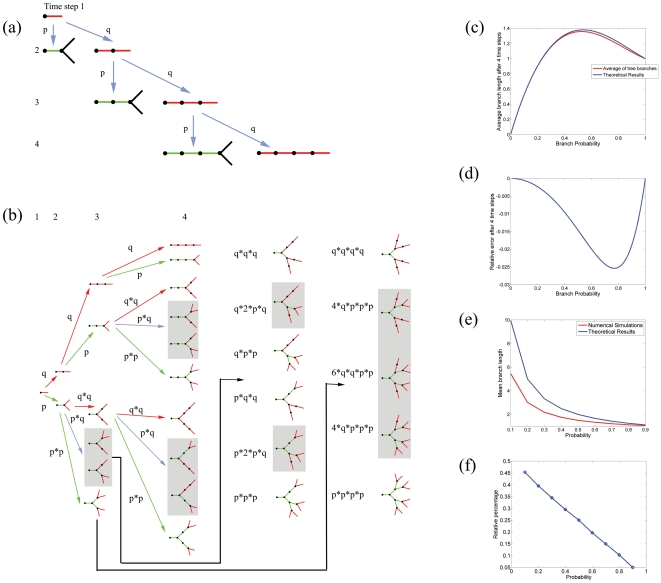
Statistics of branch dynamics observed in evolving neural trees. (a) Computing Probabilities through Single Branch Evolution Technique. After extending for one time step, an evolving neurite can undergo a branching decision in which it either branches with probability p, or extends without branching with probability q. Spawning of new daughter branches results in the termination of the parent branch. As such, at the second time step, if the neurite terminates and remains at length 2, it does so with total probability p*q. If the neurite does not branch and continues to grow, it does so with total probability q*q. As a rule, the only way that a neurite can achieve a length of n is to extend continuously for n−1 time steps and then branch. The entire sequence would therefore occur with total probability p* q^n−1^ (b) Computing Probabilities through Population Analysis of Evolved Trees. In contrast to computing probabilities of single branches as they evolve through time, a statistical analysis can be performed on instantiated trees. That is, a population distribution can be generated based on examining all possible configurations that mature (non-evolving) branches can adopt after each time step. The same probability assignments of branching with termination, and extension without branching, apply here as in (a). Note that after a few time steps, the trees start adopting non-simplistic structures. For example at t = 3, the simplest tree is a single evolving branch of length 3; which is obtained with a probability of q*q. At the opposite end of the spectrum, the most complex tree contains 4 active branches of length 1, obtained with probability p*p. Note that trees with a combination of extending and branching arbors, can occur as statistically identical configurations. Gray boxes demarcate these “isomeric” trees within all possible permutations of arbor geometries. 6 type of trees are obtained after three time steps, while 46 types of trees are obtained at the next time step. The associated probabilities can be determined by computing the products of individual probabilities along the arrows. (c) A Comparison of the Computational Results obtained from (a) and (b) at timestep t = 4. The expected value for a single branch L_average_ = (p+2*p*q) shown in blue is compared against the average branch value obtained from tree statistics (L_average tree_), shown in red, for different values of branching probability p. (d) At timestep t = 4, the relative difference (L_average tree_−L_average_)/L_average_ is plotted at different values of p. (e) Average branch values of trees obtained in numerical simulations at t = 200 (red curve) are consistently smaller than the expected values obtained from single-branch evolution. As the branching probability increases to 1, the difference between these two estimates becomes 0. (f) After t = 200 timesteps, the relative difference (L_average tree_−L_average_)/L_average_ is plotted as a function of branching probability.

After 2 time steps, the length of the elongating branch, in units of V_tip_·Δt can be described by the probability table listed in Table 1.The entries in this table are as follows: if the elongating tree branches at the first time step with probability p, the branch is considered complete and has a length of 1 (where to simplify the calculations shown here we assume without loss of generality that V_tip_ · Δt = 1). Thus, the completed distal branch (with length one) is no longer active and is replaced by two new proximally extending branches. The only way for the distal branch to increase its length is to continue to extend instead of bifurcating. This process is illustrated in Figure 2(a). If this event occurs, with probability q = 1−p, the branch will then have a length of 2. After the first 3 time steps after which two branching decisions have occurred, the table will have three entries (Table 2). The first entry remains the same, as the neurite under investigation is no longer extending, due to the branching event. The first entry does not need to be re-computed and is simply copied from previous table. The rest of the other entries, however, correspond to outcomes from an evolving branch, and as such they need to reflect the evolution of this branch. The second and third entries in the table are as follows: if the tree branches at step 2, then with compound probability q,·the tree will have length 2. As noted above, this is a terminal event for the extending branch. If the branch chooses to elongate rather than branch, with compound probability q^2^, the tree will have length 3. It is easy to check that the sum of all probabilities, (p+p·q+q^2^) = (p+q·(p·+q)) = (p+q) = 1. By induction, we can obtain the probability distribution at time step N listed in Table 3.

**Table 1:** Probability table for branches that are allowed to evolve for two time steps.

Length	1	2
Probability	p	q

In the first possible scenario, the neurite branches at the end of first timestep and cease to evolve. Its final length will be 1 and the probability of this outcome is **p**. In the second scenario, the neurite grows to a total length of two at the end of the second time step, and it has the potential to grow even further at later times. The probability of the second scenario is **q = 1−p**.

**Table 2:** Probability table for branches that are allowed to evolve for three time steps.

Length	1	2	3
Probability	p	p q	q^2^

The first entry in this table is identical to first entry of Table 1, representing a neurite that branched at the first time step and stopped evolving. The second scenario involves a neurite that branches and stops evolving after extending for two previous time steps. This event has an overall probability **p·q**. Finally, the last entry again corresponds to a neurite that grows to the largest possible extent, for a total length of three at the end of the third time step, and it has the potential to grow even further at later times. The probability of the second scenario is **q^2^**.

**Table 3:** Probability table for branches that are allowed to evolve for N time steps.

Length	1	2	3	4	…	N
Probability	P	p q	p·q^2^	p·q^3^		q^N−1^

Each entry **j = 1, 2, …, N** in the table, with the exception of the last one, corresponds to neurites that branched and stopped evolving after extending for j^th^ time. The probability for the j^th^ scenario is **p·q^j−1^**. The last entry corresponds to a neurite that has extended for N time steps and has the potential of growing even further.

If we assume that the number N has a large value, it follows that
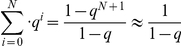
Consequently, we can use the following result:
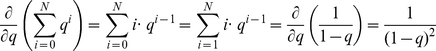
The expected value for the branch length becomes:
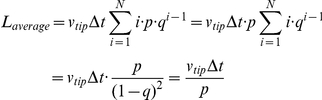



### Expected Length of Tree Branches: Statistics Derived from Formed Neural Trees

At first glance, it would seem that the probability distribution of these branches and the average branching length could be obtained from performing statistics on neural trees grown from simulations, but the overall picture is more complex. In fact, the sample mean for the branch length of the trees is a biased estimator, one which consistently underestimates L_average_.

In order to prove this point we will compare the expected branch length for trees that were allowed to evolve for 4 time steps, with the expected value for the sample mean of the tree branches. The possible instantiation of the evolving trees are shown in Figure 2(b). After the first branching decision, two types of trees are possible. The first one, obtained with probability q, contains an elongated branch of length 2; therefore, this tree cannot yet produce an estimate for the average branching length. The other one, obtained with probability p, contains one mature branch of length 1, and two evolving branches each of length 1 (for simplicity, we assume that V_tip_·Δt = 1). The latter tree has a sample mean for average branch length of 1. As a result, after the second time step (first branching decision), the expected value for the average branch length obtained from tree statistics is p*1 = p. Note that this is in agreement with the analysis in the previous section where we tracked the evolution of a single branch.

At the third time step, six types of trees are possible. These trees possess morphologies ranging from completely unbranched (probability q*q) to exuberantly branched (probability p*p) geometries. Note that it is also possible to retain trees of similar geometries using symmetry transformation. These “trees isomers” are contained within gray boxes in Figure 2(b) to indicate their identical statistical properties. Computing the sample mean for all trees results in the probability distribution described in Table 4, where we have the same convention for probabilities as the one used in Figure 2(b):

**Table 4:** Probability tables for the average branch length of trees that are allowed to evolve for three time steps.

Probability	q*q	q*p	p*q*q	p*p*q	p*q*p	1*p*p
Mature branches set	{ }	{2}	{1}	{1, 1}	{1, 1}	{1, 1, 1}
Average branch length	0	2	1	1	1	1

The entries in this table correspond to the different tree structures obtained at t = 3, as shown in Figure 2B. The first row lists the probability to obtain a certain tree, the second enumerates the length of mature branches contained in this tree and the last row displays the average branching length of these mature branches. Note that the entries 4 and 5 correspond to trees that have identical lists and averages. In particular, these trees contain an early mature branch that has generated two sub-branches. One of these sub-branches has branched again, while the other one is still evolving.

For purposes of computing the sample mean, we can consolidate the geometrically-undistinguishable structures into a single shaded entry. These tree isomers are therefore listed once, but their probability is doubled (Table 5). Based on this table, the expected value for the mean branch length is:
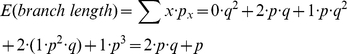



Again, this is in agreement with the results from the single branch analysis. After four time steps, however, the estimates are no longer in agreement. Using the trees generated after four time steps, we can create a table that contains the probability of generating each type of tree, the list of its mature branches, as well as the average branch length (Table 6). Since each tree can be obtained with a different probability, the average branch in each tree is a random variable described by the table above. Consequently, the expected value for the average branch in a tree is given by:
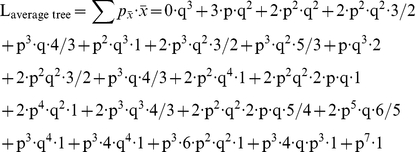



**Table 5:** Condensed probability tables for the average branch length of trees that are allowed to evolve for three time steps.

Probability	q*q	q*p	p*q*q	2*p*q*p	1*p*p
Mature branches set	{ }	{2}	{1}	{1, 1}	{1, 1, 1}
Average branch length	0	2	1	1	1

This table is identical with the previous Table 4, except that the similar trees listed in the entries 4 and 5 are now listed in the same entry (number 4), by doubling the probability of this particular scenario.

**Table 6:** Probability tables for the average branch length of trees that are allowed to evolve for four time steps.

Probability	Mature branches	Average branch length
q*q*q	{ }	0
q*q*p	{3}	3
q*p*q*q	{2}	2
2*q*p*p*q	{1, 2}	3/2
q*p*p*p	{1, 1, 2}	4/3
p*q*p*q*q	{1}	1
p*q*p*2*p*q	{1, 2}	3/2
p*q*p*q*q	{1, 2, 2}	5/3
q*p*q*q	{2}	2
2*q*p*p*q	{1, 2}	3/2
q*p*p*p	{1, 1, 2}	4/3
2*p*p*q*q*q*q	{1, 1}	1
2*p*p*q*q*2*p*q	{1, 1, 1}	1
2*p*p*q*q*p*p	{1, 1, 1, 1}	1
2*p*p*q*p*q*q	{1, 2, 1}	4/3
2*p*p*q*p*2*p*q	{1, 2, 1, 1}	5/4
2*p*p*q*p*p*p	{1, 2, 1, 1, 1}	6/5
p*p*p*q*q*q*q	{1, 1, 1}	1
p*p*p*4*q*q*q*p	{1, 1, 1, 1}	1
p*p*p*6*q*q*p*p	{1, 1, 1, 1, 1}	1
p*p*p*4*q*p*p*p	{1, 1, 1, 1, 1, 1}	1
p*p*p*p*p*p*p	{1, 1, 1, 1, 1, 1, 1}	1

The entries in this table correspond to the different tree structures shown in Figure 2B for t = 4. The structure of this table is similar to Tables 5, where again the trees with identical sets of mature branches are displayed only once, but with a correspondingly larger probability (e. g. entries 4, 10, etc).

This sum is no longer equal to the expected value for the single tree branch: L_average_ = 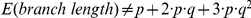, as shown numerically in the Table 7 for time step 4. This table contains a comparison between theoretical results and statistics obtained from numerical simulations of stochastically generated trees, when p = q = 1/2, for time steps ranging from 1 to 6. The probabilities and sample branch averages needed to compute the values listed above for time steps 5 and 6 have been obtained numerically, instead of by computing the sums analytically which would require a large number of terms.

**Table 7:** Comparison of average branch length resulting from single-branch and tree evolution, with probability of branching p = 1/2.

Time Step	1	2	3	4	5	6
L_a_ single branch	0	0.5	1	1.3750	1.6250	1.7813
L_a_ tree	0	0.5	1	1.3552	1.5612	1.6568
Error (percentage)	0	0	0	1.46%	4.09%	7.51%
Number of trees	1	2	6	42	1806	3263442

This comparison is done time steps ranging from 1 to 6, listed in the first row. Comparison at larger times becomes computationally prohibitive. Second row lists results obtained from theoretical considerations for the evolution of a single branch (see Table 4). Third row lists expected values resulting from the statistics of trees, (e. g. Tables 5 and 6, for t = 3 and t = 4, respectively). Computation of the entries for t = 5 and 6 has been done numerically, generating the tree trees and their corresponding probabilities automatically. Fourth row displays the relative error between these two measures, that is, (L_a__single branch_−L_a__tree_)/ L_a__single branch_. While these estimates are in agreement for small timesteps, L_a__tree_ consistently underestimates L_a__single branch_ for timesteps larger than 3. Last row displays the number of trees needed to carry out the calculations for L_a__tree_, indicating a factorial explosive growth in the number of trees required to perform the calculations for these estimates.

Furthermore, it is expected that this divergence depends on the branching probability. Indeed, in the extreme case of setting the branch probability equal to one, all branches of the tree will have the same value, hence both the theoretical prediction and statistics from numerical simulations must agree. For the trees that are allowed to evolve for 4 time steps, we can compute these values exactly (Fig. 2(c)), as well as the relative error, defined as (L_average tree_−L_average_)/L_average_ (Fig. 2(d)). The summation of resulting terms from the table of probabilities at time step 5, denoted by L_average tree_, are compared to the L _average_ = (p+2·p·q+3·p·q^2^), derived in the previous section.

We further extend the comparison between the numerical simulations and theoretical derivations of fully formed trees; that is, for trees that were allowed to evolve for 200 time steps (Fig. 2(e)). Since there is no discrepancy when p = 1 (all branches have length 1), it is not surprising that as the branching probability decreases toward lower values, these differences will increase. The results also suggest that the relative differences between these two estimates, defined as (L_average tree_−L_average_)/L_average_ , depends linearly on the branching probability (Fig. 2(f)).

Results from Table 7 suggest that as the trees are allowed to evolve, the discrepancy between the theoretical results and the sample means obtained from simulations, increases. For example, if the tree is allowed to evolve with branching probability p = ½ for 200 timesteps, we can determine the distribution of tree branches by computing the relative frequencies of the branches of different lengths. These numerical simulations suggest that the distribution of branch length obtained from tree statistics is still described by the theoretical distribution derived in the previous section (p·q^j−1^, for length j), with larger values for the ‘effective’ branching probability, equal to approximately 2/3 here, instead of the simulation value of ½ (Table 8).

**Table 8:** Comparison of single-branch theoretical predictions and statistics of mature branches resulting from numerical simulations, with p = ½ and t = 2000 time steps.

Length	1	2	3	4	5	6	7	8	9
Probability	0.5	0.25	0.125	0.0625	0.0313	0.0156	0.0078	0.0039	0.0020
Frequency	0.6608	0.2246	0.0769	0.0246	0.0090	0.0024	0.0012	0.0003	0.0000

The first 9 terms resulting from Table 4 are listed in the second row, illustrating the feature of this probability distribution (p·q^j−1^). The largest probability here, equal to 0.5, is for a branch of length 1. Each increase in length reduces the probability of the next possible outcome by q = ½. As shown in Table 7, for tree that evolve more than three timesteps, statistics of the neural tree consistently underestimate the average branching length; this corresponds to a larger effective branching probability. The probability distribution of mature branches of different length indeed seem to match a similar probability distribution of (p·q^j−1^), but with a larger effective branch probability of p≈0.66. In other words, largest probability of the last row, corresponding to a branch of length 1, is 0.66. Each increase in length reduces the probability of observing this outcome by q = 1/3.

### Naïve Prediction Without Pruning

**Figure 3:**
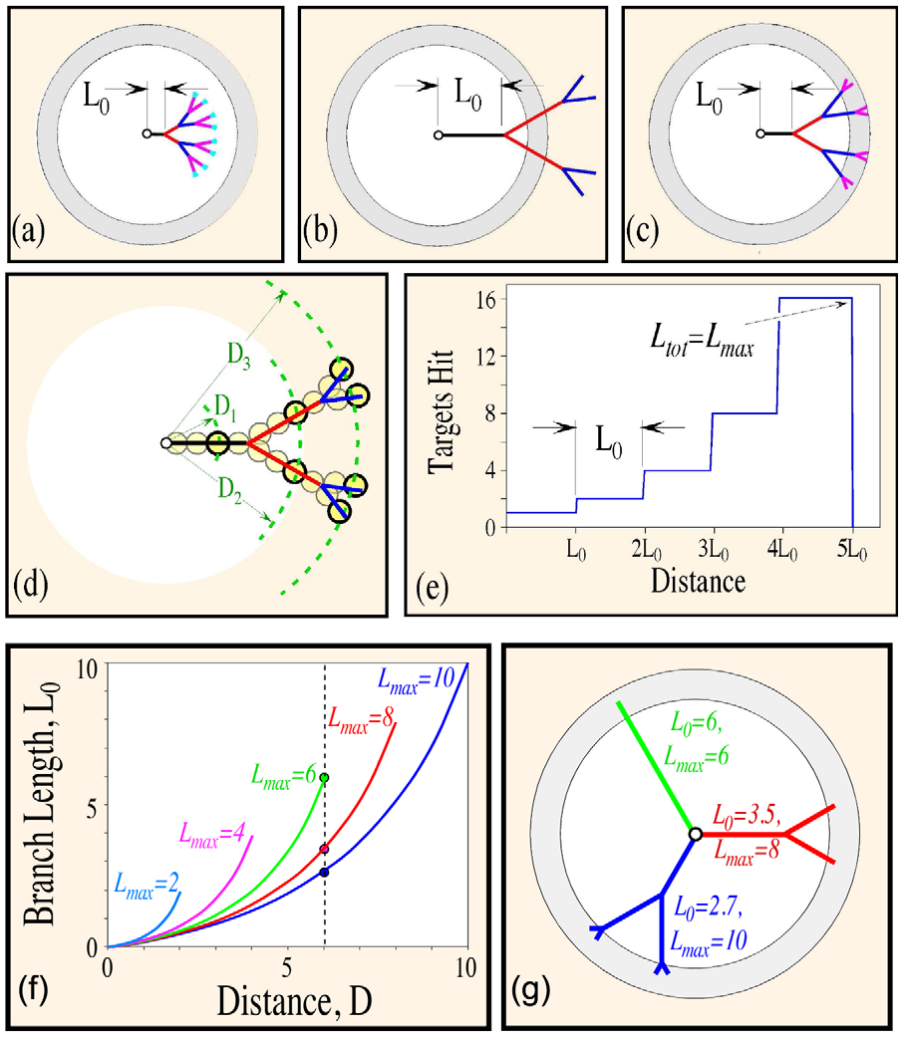
Comparison of equal length trees under different branching scenarios. (a) Undershoot, for L_o_ = 1 units and fixed cumulative neurite length L_max_; branches fail to reach targets in gray band; (b) Overshoot, for L_o_ = 4; branches reach the gray band but hit sparsely due to overextension past the target zone; (c) Optimal run, for L_o_ = 2; number of targets hit in the gray band is maximized; (d) Targets (yellow circles) that can be reached by branching neurite – note that at example distance D_1_, one target is hit; at D_2_, two are hit, and at D_3_, four are hit; (e) Number of targets hit by idealized arbor vs. distance from the origin. Once the total arbor length is L_max_, no further targets are reached. (f) Plot of the optimal branch length, L_0_, vs. the distance to the target, D, defined by Eq. [3]. For example, one can reach targets near D = 6 indicated by the dashed line in one of three ways: (1) using L_max_ = 6, (2) L_max_ = 8, or (3) L_max_ = 10. (g) Arbors for each of these three alternatives, showing that case (1) corresponds to no branches, with L_0_ = 6; (2) corresponds to a single branching event, with L_0_ = 3.5, and (3) corresponds to two branching events, with L_0_ = 2.7. Note that the arbor extends only as far as resources (i.e. L_max_) permit, and so the terminal branches are often shorter than L_0_.

For the stochastic system that we seek to investigate, each branch evolves independently. Nevertheless, by considering branches that branch regularly, we can derive expected targeting behavior for the standard or uniform neural tree.. Axons that on average branch often (depicted in Fig. 3(a) by using p = 1 and small L_0_ = V_tip_ Δt) produce bushy arbors that explore the nearby space thoroughly, but exhaust their resources rapidly and as a result cannot travel far from the originating point. Here L_0_ plays the role of the quantity L_average_ derived in the previous two sections: we assume a neurite travels exactly a length L_0_ then it branches with probability 1. Using this convention, trees that branch often will have a small L_0_, creating a dense tree, while branches that have low branching probabilities will extend for a long distance before bifurcating. As illustrated in the figure, targets located in a gray band at a fixed distance from the origin will not be reached by such an arbor. By contrast, a standard tree that branches less frequently (Fig. 3(b) e. g using p = 1 and larger L_0_ = V_tip_ Δt) could travel further from the starting point using the same resources as before, but would explore intervening space more sparsely, passing only at a couple of points through the grey target region shown. It follows that given a target distance, an intermediate branching rate (or equivalently an intermediate mean branch length, L_0_), could maximize the number of branches that explore targets at a desired distance (within the gray band in Fig. 3(c)). This example shows how maximal performance is achieved by the uniform neural tree that hits the largest number of targets at a set distance (gray area in Figure 3).

From this first order analysis, we conclude that one can choose branching rates to maximize the number of targets reached – and so the probability of reaching a particular target – at a specified distance. We note here that the standard tree with maximal targeting capabilities can still perform worse than trees with non-constant branching probabilities. For example, in the case where the target is situated far away from the starting point, a tree that evolves using a single non-branching neurite until reaching the targeting region only to branch maximally in that area, will achieve better targeting performance. From this perspective, out analysis does not select the parameters that allow an individual tree to obtain the optimal performance, but the ones which allow a class of trees to obtain on average, optimal performances.

Given a branching probability, we can use this averaged approach to derive the number of targets reached as a function of distance from the cell body in a straightforward manner. As shown in Fig. 3(d), each time a branch is produced (on average at distance L_0_ from the last branch) the number of targets hit per unit time doubles, which continues until the daughter branches travel on average L_0_ = v_tip_ Δt, at which point the number of targets hit per unit time doubles again, and so on until the cumulative branch length reaches L_max_. At the moment of the first branching, the cumulative branch length, L_total_, is L_0_; at the second branching L_total_ = L_0_+2L_0_; at the third, L_total_ = L_0_+2L_0_+4L_0_, etc. Thus, the number of targets reached grows exponentially as a function of distance from the origin as shown in Fig. 3(e) until L_max_ is reached. L_total_ itself grows according to (2^N^−1) L_0_, where N denotes the number of times the standard tree has split before it runs out of resources, which ideally occurs when L_max_ = (2^Nmax^−1)L_0_, or when N_max_ = log_2_(1+L_max_/L_o_). The condition (shown in Fig. 3(c)) that all neurite tips reach a target distance D translates to:
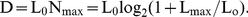
(3)Note that the implicit assumptions of equation [3] are that the neurites travel outward away from the origin, the trajectories do not curve, and the splitting angles are zero at all branching points. While these conditions are obviously not met in simulations or *in vivo*, we can use these approximations to establish an upper bound for the maximal targeting performances that can be achieved by a standard tree of fixed total length. More precisely, the upper bound for the number of targets situated at distance D for a tree of total length L_max_ is 2^Nmax−1^. Since Eq. [3] is transcendental, we show an implicit solution in Fig. 3 for various values of L_max_. For example, targeting at distance D = 6 (dashed line in Fig. 3(f)) can be achieved in a variety of ways: for a cell that is limited below L_total_ = 6 for its final total length, Eq. [3] tells us that the cell's neurite can reach the target (using a single, unbranched, axon) with L_0_ = L_max_ = 6. Alternatively (shown in Fig. 3(g)), using L_max_ = 8, the neurite can branch once and reach the target using L_0_ of about 3.5. Finally, using L_max_ = 10, the neurite can branch twice and reach the target using L_0_ of about 2.7. Thus given the resources available (defining L_max_), Eq. [3] allows us to determine the optimal branching rate (which in turn determines L_0_) needed to optimize the chance of striking a target at a given distance, D.

We reiterate that the solution shown here only presents a simplification of the neurite targeting problem. For example, actual neurite branches do not appear at constant distances and neurites do not travel in straight lines separated by fixed angles. Furthermore, when neurites do branch, their trajectories cease to be centered at the origin and the value of number of branching points N calculated above will in general not be an integer. Additionally, the simplified calculation above ignores redundant hits and finite target sizes. Nevertheless, these examples predict the behavior expected under idealized conditions: namely that the most effective standard trees should be the ones that finish the search at the required distance, defined to lowest order by Eq. [3], and not the ones that run out of resources too early or that extend too far from the origin. On average, we expect the branching rate to be such that the mean branch length, L_0_, obeys Eq. [3].

### Expected Effects Of Pruning

**Figure 4:**
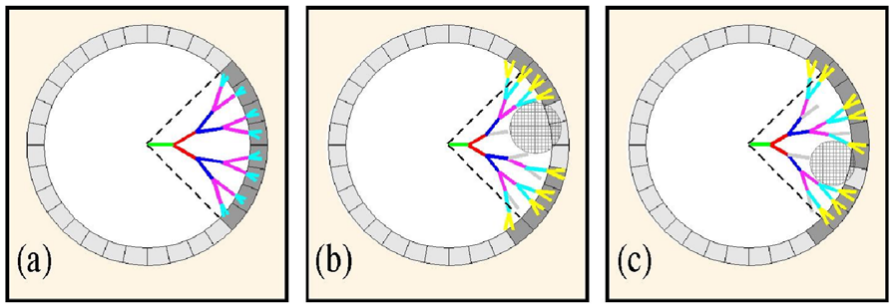
Comparison of equal length trees under different branching and pruning scenarios. (a) L_o_ = 0.9, P_prune_ = 0; (b) L_o_ = 0.7, P_prune_ = 0.25; (c) L_o_ = 0.7, P_prune_ = 0.25; L = 20, D = 2.75 in all cases. Gray branches are pruned. Note that pruned trees acheive a wider coverage of targets, extending outside of the dashed lines in panels (b) and (c); by the same token, pruning creates less uniform coverage thus canceling searches in the cross-hatched regions.

Theoretical analysis becomes more complicated when branches can be pruned. Qualitatively, pruning permits regions of space to be explored transiently after which the resource expenditure is recouped for subsequent exploration elsewhere. On the other hand, pruned branches can no longer explore downstream regions of space; and consequently, it is not at all obvious what the ultimate effects of pruning may be. To develop some insight into some of the functional effects of pruning, we provide in Fig. 4, several simplified illustrations of pruning events that we have encountered in our simulations.

In Figure 4(a), we show an unpruned tree of maximum length L_max_ = 20, with average branch length L_0_ = 0.9. The target zone is divided into targets of fixed size. In this simple example, targets to the right of the origin are uniformly struck, with 8 targets hit in total. By comparison, in Figures 4(b) and 4(c), we show trees with the same maximum length, but with higher branching probabilities balanced by a 25% pruning rate. As described previously, this means that at every 10 timesteps, 25% of free tips are selected at random for pruning, and pruned tips die back to the nearest branch point, after which no further growth is allowed.

These illustrations show that pruning permits additional space to be explored – for example, widening the coverage of targets (cf. dashed lines in Fig's. 4). Pruning, however, also produces less uniform coverage – for example, circumventing the hatched areas in Figures 4(b)–(c). Additionally, pruning reduces redundant coverage, which occurs naturally in rapidly branching trees. This depends on the size of the target: if the target size is very small, then the most important measure is the number of terminal branches and redundancy is negligible. In contrast, as target size increases, pruning can be increasingly beneficial in reducing the overall redundancy.

### Quantification of targeting efficiencies

The use of theoretical equations allows us to derive a continuous formula for the number of targets visited by the neural tree at a certain distance. For the numerical simulations, we can obtain a similar quantitative measure by defining S(r) as the number of cubes of Size 3 that are located at distance r away from origin and are being visited by the expanding neural tree. Obviously, the theoretical derivations, which constitute an upper bound for the distance where the standard tree can target efficiently, should outperform the trees obtained in numerical simulations the vast majority of the time. They always peak at the right time, just as the tree is about to run out of resources. To facilitate the comparison between theoretical and simulation methods, we consider another approach that is situated in between these models and is expected to generate intermediate targeting results. More precisely, this model allows the neural trees to branch stochastically at any moment in time, while still maintaining linear trajectories. In the upcoming sections, we use the variable S(r) to compare these three types of neural trees: standard theoretical, stochastic, and numerical.

Apparently, the effects of pruning are complex, since it is possible to obtain neural trees that extend below or above neurons generated with similar parameters but without pruning. To analyze these effects systematically, we resort to simulations. First, we examine the case of stochastic branching without pruning. Then, we turn to studying the effects of pruning on targeting, both in terms of overall targeting effectiveness and of the time required to reach the target.

### Visualization of the 3D neural structures obtained from the computational model

**Figure 5:**
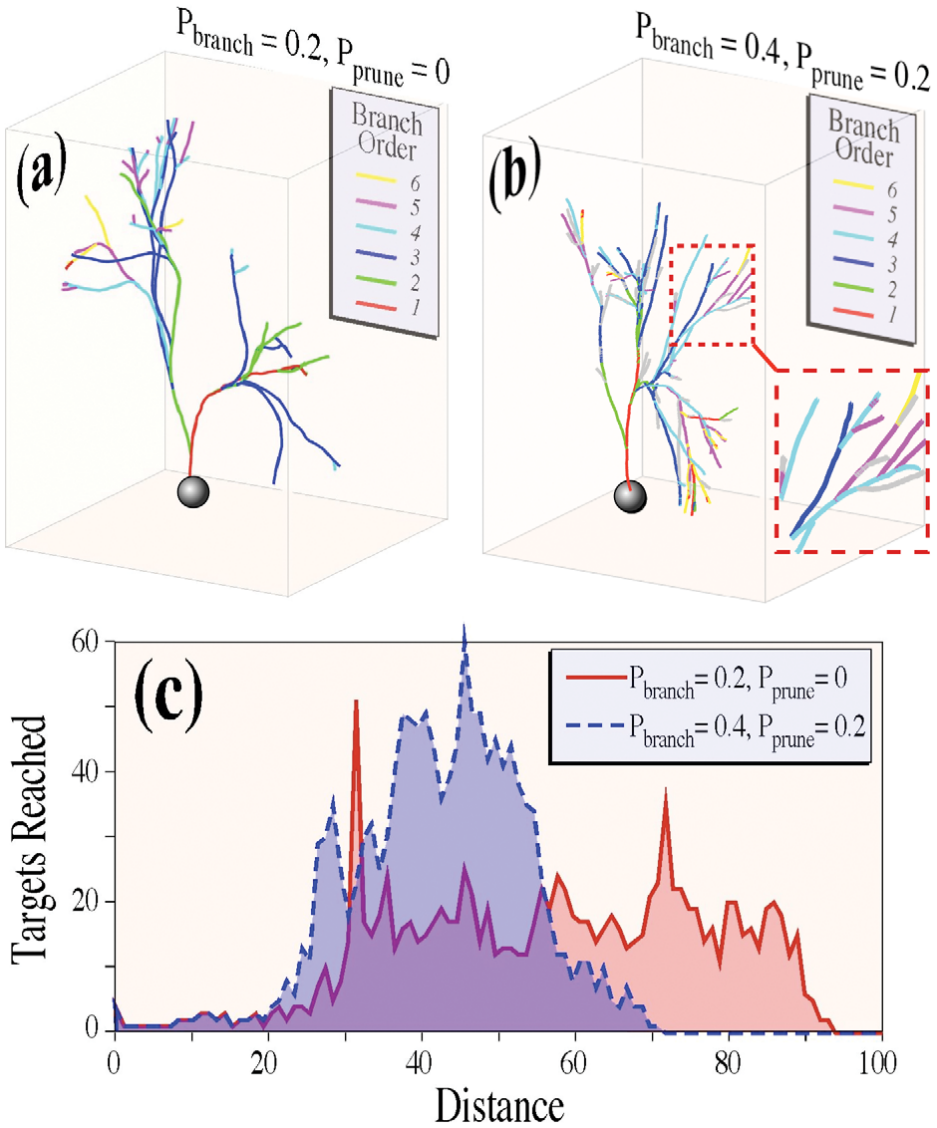
Comparison of equal length trees resulting from numerical simulations under different branching and pruning scenarios. (a) Example of a neuronal arbor generated using a computational model set at low branching probability, P_branch_, and zero pruning probability, P_prune_. Growth starts from the cell body (sphere); primary branches are shown in red, secondary branches in green, and so on as indicated in the legend. (b) A superficially similar tree with higher branching probability and a compensatory higher pruning probability. Pruned branches are plotted in gray: some examples are highlighted by the enlarged inset. (c) Plots of the numbers of potential targets reached as a function of distance away from origin quantifies the trends exhibited by the neural trees from the previous panels, showing that similar-looking trees can exhibit significantly different targeting capabilities. In these examples, the pruned state predominantly reaches shorter distances, yet strikes 15% more targets overall than the unpruned case.

As mentioned in the methods section, we use an arborization model to examine complex structures of axons observed in sensory neurons with the goal of exploring the implications of axonal morphological variations on target-finding capabilities. In particular, we focus here on examining the effects of pruning – i.e. elimination of some projections – on targeting. As an illustration, we compare two typical simulated neuronal arbors in Figure 5. In brief, neurite tips wander stochastically in 3D, and each free neurite tip has one fixed probability, P_branch_, of branching into two free tips in a unit time, and a second fixed probability, P_prune_, of being pruned and dying back to the nearest branch point in the same unit time.

In a first scenario, shown in Figure 5(a), neurite tips branch with probability P_branch_ = 0.2, meaning that an average of 20% of free tips, chosen at random, will branch at constant time intervals, but P_prune_ = 0, meaning that branches never prune. By comparison, in Figure 5(b), neurites branch and prune more often, with P_branch_ = 0.4, and P_prune_ = 0.2. After 130 computational time units, the two simulated neural arbors are superficially similar, but as shown in Figure 5(c), exhibit significantly different rates of striking targets with constant volume, S(r), at given distances from the originating points.

In these simulations, we assume that resources available to cells are limited, which we mimic by imposing the constraint that the total length of the neuronal arbor cannot exceed a maximum. The total arbor lengths are therefore are identical in Figures 5(a) and 5(b). Despite this constraint, it is apparent in this example that the simulation without pruning reaches targets further from the origin than does the simulation with pruning. We remark that although this result is typical, it is not universal. Due to stochastic variations, there exists a similarly large number of simulations in which a tree that is pruned may nevertheless reach more distant targets than an unpruned tree. We present statistics for multiple replicates in subsequent sections. On the other hand, by integrating over the total number of targets hit, we find that the simulation without pruning reaches 15% fewer targets overall than the simulation with pruning. This increase due to pruning constitutes a general trend for all simulations. In the following sections, we use large-scale statistics obtained from multiple simulations to quantify the targeting efficiency as a function of distance away from the starting point.

### Simulations without pruning

**Figure 6:**
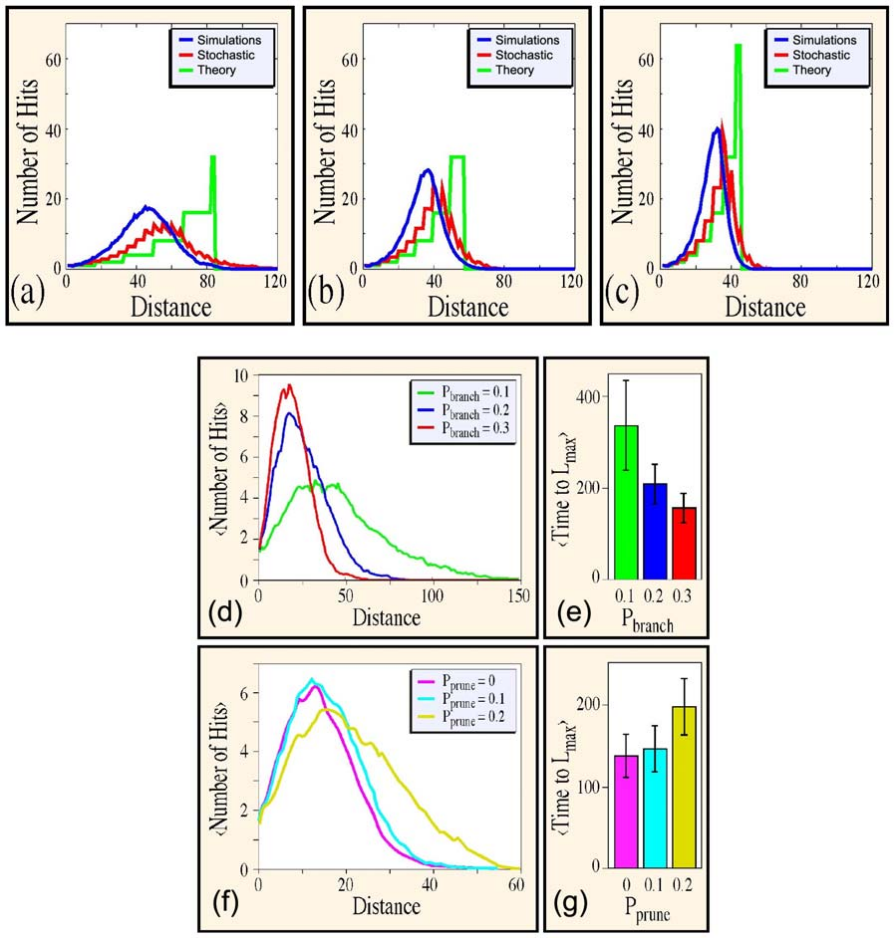
Estimates for the number of hits at a distance D for evolving neurons. Cases shown use the following branching rates in (a) P_branch_ = 0.3; (b) P_branch_ = 0.5; (c) P_branch_ = 0.7. All these three panels show results from (i) full-fledged numerical simulation (blue, averaged over 100 runs), (ii) simplified trees that have a stochastic branching time (red, where the time intervals between branching decision does not have a fixed length), and (iii) from theoretical considerations (green). As expected, the use of lower branching probabilities reduces the number of hits at smaller distances, but allows reaching targets that are further away. The position of the ‘optimal’ targeting distance for distinct branching probabilities is in qualitative agreement over the range of probabilities considered here. Note that the stochastic (red) and theoretical (green) curves both have discontinuous first derivatives, in contrast to the numerical (blue) curves. (d) Statistical results for neural trees with branching or pruning for L_max_ = 1000 units. Plots of targeting rates from numerical simulations indicate that neurites with low probabilities of branching reach further, but fill less surrounding space. (e) A complementary result is that at low probabilities for branching, it takes longer for a neuron to exhaust its resources and reach the maximum allowable arbor length, L_max_. (f) Neural trees that have been generated at higher pruning probabilities reach further from the origin and (g) take more time to finalize the ultimate arbor. We note that P_branch_ = 0.3 in panels c and d.

When branches prune stochastically, rather than at fixed uniform intervals, the targeting distribution changes significantly. Rarely does the majority of branches reach higher order arborization simultaneously. Consequently, the dominant peak at long distances is considerably diminished in favor of a single mode at a moderate distance. We can estimate how stochastic variations in branching should affect targeting simply by repeating the calculation made in Figure 3(e) numerous times, permitting each branch to form at a different time, according to a Poisson probability distribution. Doing so for 100 simulations each, we produce the plots shown red in Figure 6 ((a) to (c)) for P_branch_ = 0.3, 0.5, and 0.7, which as discussed are unimodal, with a peak at moderate distance, rather than at the maximum distance shown in green for the uniformly branching case. Contributing to the spreading of the distribution results is the existence of some rare events in which branching seldom occurs, and the tree can reach far beyond the maximum distance defined by Eq. [3]. The red and the green plots, shown for three values of branching probability in Figure 6 ((a) to (c)), are to be compared with full simulations, again for 100 trials, as described in the Methods section. These results are shown in blue in these figures. In the full simulations, we subdivide the entire computational domain into volume elements 3 distance units on a side, and define the number of targets hit to be the number of voxels penetrated by at least one neurite tip. Throughout our analysis, a hit is recorded whether or not the relevant tip is subsequently pruned.

As we have mentioned previously, the theoretical (green) and stochastic (red) results are approximations of the simulation conditions, neglecting neurite curvature, angle variations at branching, redundant branches, finite target size, etc. As a consequence of these approximations, optimal targeting occurs at lower distances in the simulations (blue line). Nevertheless, both approximation cases reveal the essential mechanism at work in targeting when branching is also manifest in the simulation results. More explicitly, the region of optimal targeting shifts to locations that are situated farther away as the branching probability decreases. As demonstrated by the numerical simulations, exponentially more targets are reached by higher order than by lower order branches. The relative paucity of very high order branches, however, as well as the existence of occasional trees that reach long distances, combine to produce smoother targeting curves with a single maximum performance peak. In the presence of pruning, this result changes significantly, as we describe next.

### Simulated effects of pruning

The previous results demonstrate the intuitive result that standard trees which branch more often can travel shorter distances, but cover the intermediate range better than do branches that bifurcate less frequently. A quantitative summary of these results appears in Figure 6(d). A corollary is that the more branches present, the more rapidly a neuron will exhaust its resources and need to stop searching for targets. This is shown in Figure 6(e), where we plot the time, averaged over 500 trials, to reach a maximum total arbor length L_max_ = 1000. Accordingly, we expect that an increase in pruning probabilities should reduce the effective rate of producing new branches, thus increasing the range at which a standard tree can search for targets, as well as the overall search time. Numerical simulations are in agreement with this intuition, as indicated by results with P_branch_ fixed at 30% and varying P_prune_, which are shown in Figures 6(f) and 6(g). The results of Figures 6((d) to (g)) indicate that branching and pruning can be tuned to maximize rates of striking targets as functions either of time or of distance. We carry out more systematic numerical simulations, presented in the next section, that confirm this conclusion.

### Branching and pruning – results from numerical simulations

**Figure 7:**
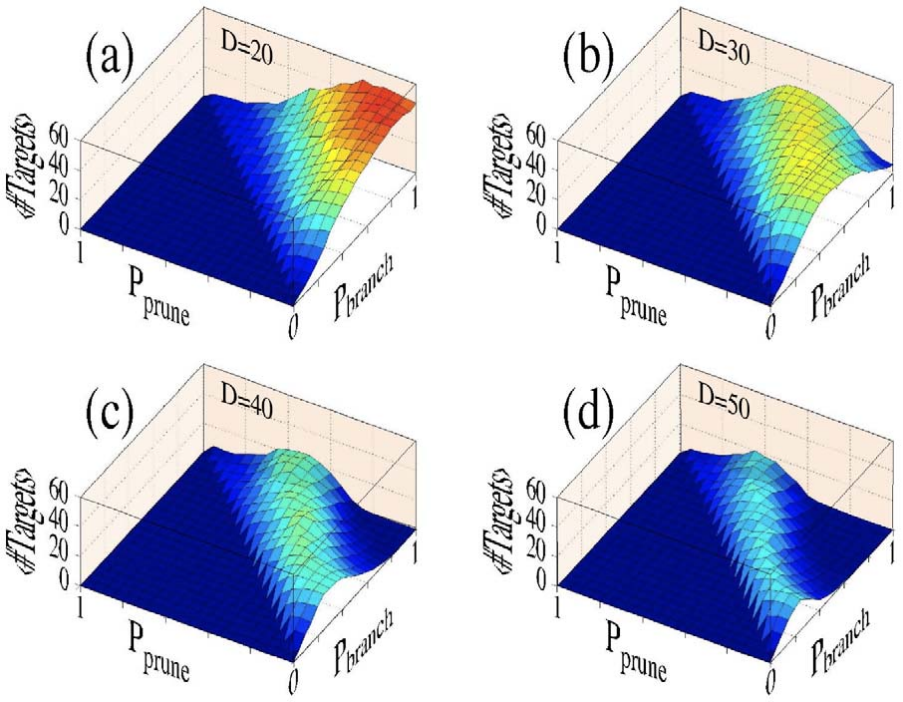
Number of targets reached vs. branching and pruning rates. The following parameters have been used to generate these panels: (a) D = 20; (b) D = 30; (c) D = 40; (d) D = 50. For potential targets close to the cell body (case (a)), the most effective strategy is to branch as much as possible while pruning as little as possible, while for more distant targets, pruning rates must increase with branching rates to provide high targeting probabilities – i.e. pruning is required to provide optimal targeting.

The theoretical considerations that we have described indicate that optimal branching and pruning rates can be established to construct standard trees that maximize probabilities of striking particular targets. To confirm these results, we perform a factorial design of simulations in which branching and pruning rates are varied, and the number of fixed size targets is evaluated as a function of distance to the target. Results are shown in Figure 7 for distances D = 20, 30, 40 and 50. These results have been obtained for 500 repetitions of simulations where P_branch_ = 0, 0.1, 0.2, …, 1, and P_prune_ = 0, 0.1, 0.2, …, 1. Note here that the number of simulations have been increased from 100 to 500 to allow for more statistical confidence in comparison of performances for adjacent regions of the map. The target size is 3 units on each dimension. In addition, simulations are performed only if P_branch_−P_prune_>0, that is, the targeting success is deemed to be zero if P_branch_−P_prune_≤0.

Figure 7 indicates the existence of two trends. First, maximal targeting rates are produced in all cases along directions that start parallel to the diagonal, where P_branch_−P_prune_ is constant. Deviations from the expected parallel line can be observed in numerical simulations for high values of P_branch_ and P_prune_, due to the increased incidence of accidental imbalances in local branching and pruning events. Second, for nearby targets (e.g. Fig. 7(a)), P_branch_−P_prune_ is large, meaning that reaching such targets requires a maximal branching rate and no pruning, while for intermediate ranges (e. g. Fig. 7(b) and 7(c)) balanced branching and pruning is needed for optimal targeting. Note that for distant targets (e.g. Fig. 7(d)), P_branch_−P_prune_ is small, meaning that in order to reach these targets, one must prune aggressively even when the branching rates are not set at high values. In principle, even for values of P_branch_−P_prune_ that are not too small, it is possible to completely prune a tree by accident. In order to prevent entirely denuding a tree in our simulations, we implemented a rule that a tree with only one branch left would not be pruned.

We note here that our findings directly show that optimal targeting for a set distance can be achieved in the complete absence of pruning. However, trees that prune in effect permit additional target searches at intermediate distances, a feature that would be useful to increase target efficiency for a range of distances (as opposed to a single fixed distance). This represents an advantage that trees which prune have over trees that only branch, thus pointing to potential functional roles of pruning. We note, however, that by operating in a high branching/pruning regime, a cost incurs due to an increased likelihood that putative successful branches might be prematurely cut off or that the tree may waste its resources on premature growth if the pruning is deficient at early stages. In this sense, a moderate amount of pruning would strike a balance between too little intermediate exploration and too many premature pruning events. We also note that by comparing trees which have similar total length for their final structures, we implicitly assume that there is no penalty for pruning of neurites. This simplification permits us to perform an explicit analytical treatment. Different options for assessing cost can be employed in higher order models, such as fixed terms, factors that are proportional to the length of the neurite pruned or factors that depend on the distance from the starting point.

Results from the numerical simulations support the notion that branching and pruning may work as opposing agents to collectively help a neural tree find its target. In other words, as long as there is a set difference between probabilities of branching and pruning (P_branch−P_prune), the expected targeting success at a distance D away from the origin should be similar. To further explore this, we recognized that the search for targets at a certain distance from the initial starting point can be formulated as an optimization problem, in which a neural tree attempts to advantageously use the limited resources by generating a structure that maximizes the number of active neurites in the region of interest. We used theoretical methods to generate an upper bound estimate for the target performances for neurons that have a fixed probability of branching and a fixed total length, showing that the tree optimally targets at a specific distance.

**Figure 8:**
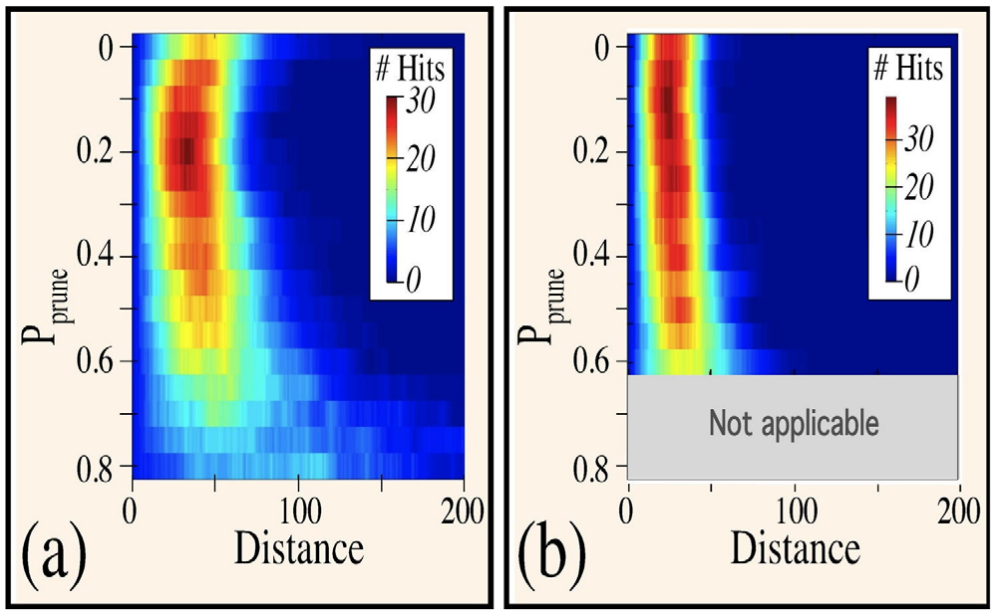
Colorscaled plots of targeting performances as a function of distance. Here the difference between branching and pruning probabilities is kept constant at (a) 0.2 and (b) 0.4. In both panels, the maximum value for P_branch_ = 1. As anticipated from Fig. 2 and confirmed by Fig. 6, smaller P_branch_−P_prune_ – i.e. more frequent pruning – is more effective at reaching distant targets.

While the requirement that a fixed difference between branching and pruning rates is indicative of the optimal targeting distances, these trends may exhibit some variations. We can investigate this directly by examining the effect of pruning on targeting as a function of distance to the target by fixing P_branch_−P_prune_ and plotting the number of targets hit versus P_prune_ and distance. As shown in Figure 8(a), close examination of the overall performances as a function of distance when the difference between branching and pruning probabilities is relatively low (P_branch_−P_prune_ = 0.2) indicates that trees generated under this parameter regime are most effective at targeting at distances around D = 50. While in general, the performances are similar for different parameters, trees that have very high branching and pruning probabilities loose their effectiveness at targeting at this distance. Hence, Figure 8(a) shows that for P_prune_>60% and P_branch_>80%, few targets are reached at any distance. This appears to be associated with the fundamental exponential nature of iterative branching. That is, at high branching rates, the number of branches along with total arbor length grows exponentially in time. A small stochastic reduction in pruning can therefore cause a large growth in total arbor length and a premature exhaustion of available resources. On the other end of the spectrum, a tree that prunes overeagerly by a small amount produces slender and sparse arbors that make inefficient use of the resources in the intended target area. In contrast, as shown in Fig. 8(b), trees generated with a larger difference between branching and pruning probabilities are better at targeting at smaller distances and are relatively more robust in the high branching/high pruning regime.

## Discussion

### Landscape profiling is useful in evaluating target-finding strategies

In this paper we have constructed a computational model of a growing neuron with stochastic capabilities to evolve individual branches. The growth dynamics within the simulations were adjusted according to observations of sensory neurons isolated from the dorsal root ganglion (DRG) and grown on a featureless growth-permissive substrate. Live cell imaging demonstrates that neurons dramatically restructure their arbor architecture as they grow in culture (Fig. 1). These processes, such as branch formation and branch elimination, were therefore integrated into our *in silico* model to reflect the complex arborization morphology of DRG neurons observed *in vitro*. While individual branches were allowed to evolve independently, a length-bound scenario was assumed to realistically reflect the finite amount of cellular structures that can be sustained by a neuron as it expands. In contrast to the traditional method of estimating targeting success by employing a single stationary target, we mapped the entire search space covered by the neural trees. The resulting “landscape profiles” obtained for neurons with differing pruning and branching capabilities allow for a more informative measure of how arborization dynamics change the topography of the space searched and consequently, the ability for a neuron to innervate a potential target located within those coordinates.

Our work is different from the traditional search and the pursuit problems in the sense that the target does not attempt to evade the search. Instead, they are hidden at the beginning of the search. Although there are similarities with complex search problems (e. g. [3]), the timing of branching and pruning induces complications by creating multiple search locations per trial. Some weak cues may exist in the environment, but here we consider the behavior emerging from a neural targeting model that has a small amount of assumptions. This allows us to characterize the expected targeting performances of the model using theoretical and computational results, and provide the framework for incorporating additional refinements, such as the ones related to the chemical cues, as they become available from future experiments.

At the initial stages of the computational modeling, the parameters have been tuned to give rise to realistic-looking neurons that resemble the morphometric studies of the *in-vitro* neurons. Since similar-looking neurons can be obtained over a range of parameters (see Figures 4 and 5 for examples of branching and pruning parameters), the focus of our simulations is to explore the behavior of classes of neurons and to examine if they exhibit useful targeting behaviors. Examination of evolutionary behavior rather than final shape, avoids the issue of over-fitting the details of the final arborization structure, and instead focuses on examining the dynamics of neural structures that emerge from realistic growth rules.

Our work complements previous findings in optimal neural wiring [25]–[29] that show how the topology of neural tissue in more compact areas, such as the brain hippocampus or cortex, is influenced by local branching rules. In contrast, the phenomena described here are related to spinal cord injury regeneration research, where phenomena not normally occurring in human body, namely re-establishment of ascending and descending the neural pathways, are the desired outcomes of research.

### Pruning is an active contributor rather than passive consequence of target finding

Of particular interest is the influence of branch elimination, or pruning, on the landscape profile of arborization neurons. Branch elimination is crucial in the refinement and resculpting of axons to ensure proper formation of functional circuitry (reviewed in [10], [31]). Axonal pruning plays a key role in removing superfluous or misguided branches in developing vertebrate nervous systems: ectopic branches of retinal ganglion cell axons in retino-tectal map development [32], redundant axonal inputs of terminal arbors at synapses of developing neuromuscular junctions [33], exuberant cerebrocerebellar collaterals in neonatal cat brains [34]. Process elimination has been observed at different scales ranging from fine-tuned pruning of dendritic spines in the neocotex [35]–[36] to large-scale elimination of the axon collaterals of major cortical projections, characterized by rapid pruning of many millimeters of primary arbor including its distal higher-order branching structures [9]. Axon elimination does not require commitant synapse disassembly, as pruning can occur in the absence of synapse formation [8], [10]. Although the selective ablation of excessive or aberrant neurite extensions are events critical to developmental brain plasticity, the pruning process has been incorporated in only a handful of computational modeling studies of growing neurons in the context of formation of retinotopic maps in the brain (e. g. [37]–[38]). Our present model implements this pruning process, enabling us to generate realistically-looking neurons and to evaluate alternative search strategies in target-finding.

### A combined Branching and Pruning strategy is more efficient for targeting than a simple Branching strategy

Our results suggest that moderate amount pruning can be beneficial for the search process. By abandoning the search in certain areas after a certain terrain has been covered, the neural tree can focus on other regions, thus increasing the chance of success. As long as the neural tree does not operate in a high branch/high prune regime, which may induce too many errors due to fluctuations, pruning allows the tree to cover more ground, especially at intermediary stages, thus rendering the search more effective. Landscape profiling from the simulations shows that pruning enables a neural tree to cover larger amounts of space, but at the expense of detailed exploration of mid-range terrain. Although small targets positioned at intermediate distances may be overlooked, neurites are able to seek targets placed at long-range distal fields. This particular approach would be useful as a regeneration strategy following trauma to the adult peripheral sensory nervous system where the target of innervation for the PNS branch of the DRG sensory neuron is positioned at a far distance from the spinal cord. Regardless of the particular subpopulation of DRG neuron that conveys the distinct peripheral stimuli associated with a particular somatosensory modality, most axonal inputs from the periphery share the commonality of traversing long distances. Proprioceptive projections carry signals from muscle while mechanoreceptive and nociceptive projections carry signals from skin. Because adult peripheral axons demonstrate plasticity after injury [39], the capacity to prune may constitute an intrinsic capability for a sensory neuron to optimize its search for peripheral targets. The propensity of peripheral nerve processes to restructure its arbors provides impetus for identifying endogenous cell factors that promote pruning. Semaphorin 3F, for example, has been implicated in pruning during hippocampal development [40]. Furthermore, the growth-permissive nature of the post-trauma PNS environment [41]–[42] suggests that there may be extracellular factors which block or activate cell signaling cascades associated with the pruning process. Exciting studies reveal that glial cells themselves can actively participate in the execution of the pruning process and the engulfment of degenerating axon fragments termed “axosomes” [43]–[44].

### *In silico* results predict the existence of *in vivo* pruning factors and mechanisms which complement branching factors in directed search strategies

To date, much progress has been made in uncovering the intrinsic programs and extrinsic factors that control branch formation. Results from the numerical simulations support the notion that branching and pruning may work as opposing agents to collectively help a neural tree find its target. The active participation of pruning within positive *in silico* outcomes underscores the importance of determining factors that govern branch elimination *in vitro*. Several “branching factors” such as the Slit/Robo signaling system [45]–[46] along with other strong candidates involved in arbor formation such as Sema 3A, Anosmin, B class ephins, and Wnts [32], [47]–[48] have been closely examined; however, there is a paucity of research surrounding “pruning factors” and the mechanisms through which branch elimination is regulated. An equivalent understanding of pruning would provide insight into how pruning factors may work in concert with branching factors to direct arborization and establishing appropriate branched morphologies in developing neurons. Identification of pruning factors and associated pathways of regulation would also provide a valuable addition to the toolbox of regenerative approach

Identification of pruning factors and associated pathways of regulation would also provide a valuable addition to the toolbox of regenerative approaches, with particular applicability to scenarios where functional recovery relies on effective target re-inervation at distant loci. Moreover, isolation of pruning factors would enable their use as “correction agents” to edit mistakes that may occur during directed regrowth. For instance, redundant or aberrant extensions can be selectively ablated by manipulating the microenvironment at either intermediate branch points or at terminal arbors. This additional level of safety compensates for situations where regrowth goes awry, enabling the coveted property of fault-tolerance to be engineered into potential strategies used in regeneration. For example, in retinotectal targeting in mammals, it is well known that branching and searching for targets does not initiate until advancing axons reach an appropriate domain in the tectum, after which branching proliferates [49]. Such an arbor in which branching is delayed, or other arbors in which pruning depends on distance from the origin, length or order of branches, have previously been described, for example in [50]. The effect of these modifications to the pruning rules is one of the future directions for our research.

### Maintaining a fixed difference (P_branch_−P_prune_) is more important than the independent values of P_prune_ and P_branch_

Although both branching and pruning influence the landscape profiles, the most efficient search strategy is the one which maintains a balance between branching and pruning probabilities. These theoretical estimates constitute a reductionist's approach in representing the actual target-search problem; however, the resulting predictions are in agreement with the landscape profiles generated from the full-scale computer simulations. Maintaining a constant difference between branch formation and elimination gives rise to a class of neurons generated using the same set of parameters that achieve optimal targeting at the same distance. The significance of this numerical difference, has been demonstrated both by theoretical treatment and by numerical simulations, in which shifts in optimal target distances, that is, the distances at which the number of active searching neurites are maximal, are observed for neuron families of different branching and prune rates.

These findings indicate that absolute pruning and branching probabilities are less relevant to a neuron, than its capability to sustain a tightly coupled relationship between its branching and pruning probabilities (P_branch_−P_prune_). For example, if a neuron is stimulated by external factors to branch, it may recover the optimality of search by increasing its internal propensity to prune. The number of possible (branch, prune) combinations that exist for successful targeting also suggests that branching and pruning do not function as independent processes within an optimized search strategy. For any branching rate, a pruning rate exists to match and to create the appropriate balance to facilitate efficient search. A reasonable way for a neuron to achieve balance is by employing a coupling factor that operates on a higher level of control, simultaneously regulating both pruning factors and branching factors to maintain the critical difference, or constant delta. As revealed by the numerical simulations in this study, the importance of a balanced P_branch_−P_prune_ rate, which allows families/classes of neurons generated using same set of parameters to optimally target at a specific set distance away from the origin, suggests that a regulatory hierarchy for control of arbor formation may exist within the neuron.

Our current model evaluates landscape profiles generated for all (branch, prune) pairs in which P_branch_−P_prune_>0. For the simulation to evolve it is imperative that P_prune_ not exceed P_branch_, as larger P_prune_ values result in a null-growth neuron. A regulatory factor would play a role not only in maintaining the optimal difference between P_prune_ and P_branch_, but also in ensuring that P_prune_ is kept smaller than P_branch_. These implications assume a constant P_prune_ is maintained throughout the evolution of the neural tree. It is possibility for P_prune_ to exceed P_branch_ at certain timepoints, while maintaining an average P_prune_ that is less than P_branch_. How sporadic increases in P_prune_ beyond P_branch_ influences search potential, remains to be determined. A possible approach is to decouple P_branch_ from P_prune_ and investigate the effect of varying P_prune_ values within the simulation of a single neural tree. Of particular interest are search strategies associated with parametric-dependent pruning. In addition to evaluating strategies which employ dynamical pruning and branching rates, future work will involve expanding the current neural growth model to include neutrophic factors, chemo-repellent and attractive spot guidance cues, as well as gradient cues. These elaborated models will provide further insight into how neurons can be directed to regrow and innervate targets within physiologically-relevant scenarios.
